# Melon Peel Incorporation in Soy Protein–Maize Starch Extrudates: Effects of Formulation and Extrusion Variables on Chemical Composition, Antioxidant and Sensory Properties

**DOI:** 10.1155/ijfo/2197550

**Published:** 2026-09-06

**Authors:** Betsabé Hernández-Santos, Jesús Rodríguez-Miranda, Juan G. Torruco-Uco, José M. Juárez-Barrientos, Cecilia E. Martínez-Sánchez, José M. Rivadeneyra-Rodríguez, Andrea Ángeles-Hernández, Enrique Ramírez-Figueroa

**Affiliations:** ^1^ National Technological of Mexico/Technological Institute of Tuxtepec, San Juan Bautista Tuxtepec, Oaxaca, Mexico; ^2^ Institute of Agricultural Engineering, University of Papaloapan, Loma Bonita Campus, Loma Bonita, Oaxaca, Mexico, unpa.edu.mx

**Keywords:** antioxidant activity, dietary fibre, extrusion cooking, functional snacks, melon peel, response surface methodology

## Abstract

The aim of this study was to evaluate the effect of extrusion conditions and formulation on the physicochemical, antioxidant, colour and sensory properties of extruded snacks enriched with melon peel (MP). For this purpose, response surface methodology (RSM) was applied using an experimental design in which the independent variables were barrel temperature (BT: 120°C–170°C), feed moisture content (MC: 16–25 g/100 g), soy protein concentrate/maize starch (SPC/MS: 25–40 g/100 g) ratio and MP (10–25/100 g) content. The response variables included chemical composition (moisture, ash, protein, fat, fibre and carbohydrates), colour parameters (*L* ^∗^, *a* ^∗^ and *b* ^∗^), antioxidant activity (DPPH and ABTS) and sensory attributes (aroma, colour, flavour, texture, appearance and overall acceptability). The results indicated that increasing MP content enhanced dietary fibre levels and antioxidant capacity; however, high levels of this by‐product tended to produce denser structures and to reduce the expansion index of the extrudates. Feed MC was identified as the process factor with the greatest influence on the physical and sensory properties, as it regulates melt viscosity and the development of the expanded structure during extrusion. In addition, increasing extrusion temperature promoted browning reactions that altered colour parameters and contributed to the formation of compounds with antioxidant activity. Intermediate levels of MC and MP, combined with moderate‐to‐high extrusion temperatures, produced extrudates with a better balance between nutritional value, technological properties and sensory acceptability. Therefore, the incorporation of MP represents a viable strategy for the valorisation of agroindustrial by‐products and the development of extruded snacks.

## 1. Introduction

Consumer demand for healthier, more sustainable food options has pushed the food industry to invest, over the past several years, in extruded snacks with improved nutritional and functional value. Amongst processing technologies, thermoplastic extrusion stands out for producing expanded cereals and ready‐to‐eat snacks, owing to its versatility, its energy efficiency and its capacity to modify the physicochemical and structural characteristics of food materials [1]. As the material moves through the extruder, the joint action of heat, pressure, shear and moisture drives major changes within the food matrix, gelatinising starch, denaturing protein and generating the cellular structure that gives extruded products their characteristic crunch [2].

Conventional extruded snacks rely mostly on starch‐rich flours, such as maize, rice or wheat, which yield products high in carbohydrates but nutritionally limited. To address this shortfall, research has increasingly focused on fortifying such snacks with functional ingredients or agroindustrial by‐products capable of improving their nutritional value whilst supporting a more sustainable food system [3]. Residues left over from processing fruits and vegetables are especially relevant in this regard, as they supply substantial amounts of dietary fibre, phenolic compounds and other bioactive metabolites that can be worked into extruded formulations to boost their functional properties [4].

Recent studies have demonstrated that the incorporation of fruit and vegetable by‐products into extruded formulations can significantly improve the nutritional and functional quality of snack products. For instance, the incorporation of mango peel, apple pomace, citrus pomace, pineapple by‐products and legume‐based fibre sources has been associated with increases in dietary fibre, antioxidant activity, mineral content and bioactive compounds in expanded extrudates. However, these ingredients may also modify melt rheology, expansion behaviour, texture (TEX) development and colour (CO) formation due to their interactions with starch and protein components during extrusion processing [3, 4].

Furthermore, previous research has demonstrated that extrusion variables such as feed moisture, barrel temperature (BT) and formulation composition strongly influence the structural and physicochemical properties of fibre‐enriched extrudates. Starch dilution caused by increasing fibre or protein incorporation may alter starch gelatinisation, bubble growth, melt viscosity and cellular structure formation, thereby affecting expansion and sensory quality of the final product. Consequently, optimisation of both formulation and extrusion conditions is essential for obtaining nutritionally enriched snacks with acceptable technological and sensory properties.

Because of this, *Cucumis melo* L. peel has drawn growing research interest as a fibre source: It is rich in dietary fibre, minerals and antioxidant compounds that can enhance the nutritional value of the foods into which it is incorporated. Prior work indicates that fibre‐rich by‐products of this kind, once included in an extruded matrix, can reshape the product′s microstructure, degree of expansion and TEX, largely through their interaction with the surrounding starch–protein network during processing [5].

In addition to its nutritional composition, melon peel (MP) may actively influence the physicochemical behaviour of starch–protein systems during extrusion processing. The high dietary fibre content can interfere with starch gelatinisation and bubble growth, thereby affecting melt viscosity, expansion behaviour, TEX and density of the final extrudates. Simultaneously, interactions between soy proteins, starch polymers and fibre components may alter the structural development of the extruded matrix through hydrophobic interactions, hydrogen bonding and competition for water availability. Furthermore, phenolic compounds naturally present in MP may contribute to antioxidant activity and participate in Maillard‐type reactions during extrusion, influencing both CO development and sensory perception of the final product.

However, the incorporation of these ingredients may also affect the sensory and technological characteristics of the product; therefore, it is necessary to optimise both the formulation and the processing conditions simultaneously.

Response surface methodology (RSM) has been widely established as an effective statistical tool for evaluating the combined effects of multiple process and formulation variables in complex food systems. This approach allows different quality responses to be modelled and optimised by analysing the interactions between experimental factors, thereby facilitating the identification of operating conditions that maximise the technological, nutritional and sensory properties of the product simultaneously [6]. Even so, work specifically addressing MP within soy protein–maize starch extrusion systems remains scarce, and little is known about how simultaneously optimising the nutritional, antioxidant, technological and sensory responses is affected by different extrusion conditions when this by‐product is used. The way MP incorporation and protein–starch interactions jointly shape extrusion behaviour and final product quality is likewise still poorly understood. Building on this gap, the present study set out to evaluate how BT, feed moisture content (MC), the soy protein concentrate/maize starch (SPC/MS) ratio and MP content affect the physicochemical, antioxidant, CO and sensory properties of extruded snacks, using RSM as the analytical framework. The resulting information is intended to support the development of extruded snacks that combine improved nutritional profiles, suitable technological behaviour and strong sensory acceptance, whilst advancing the valorisation of agroindustrial by‐products and the design of sustainable functional foods.

Although previous studies have evaluated the incorporation of fruit and vegetable by‐products into cereal‐based extrudates, limited information is available regarding the simultaneous effects of MP incorporation and extrusion variables on the physicochemical, antioxidant, CO and sensory properties of soy protein–maize starch extruded systems. Moreover, the combined interactions between MP fibre, soy protein concentrate and extrusion processing conditions have not been comprehensively evaluated through RSM. Therefore, the novelty of the present study lies in the integrated optimisation of formulation and extrusion variables to develop nutritionally enriched extruded snacks with improved functional and sensory properties whilst promoting the valorisation of melon‐processing by‐products within a sustainable food production approach. This manuscript is a companion study to Hernández‐Santos et al. [7], derived from the same central composite design and the same set of 30 extrusion trials. The two studies, however, pursue distinct research questions and are not duplicative. The companion paper characterised the process‐engineering and structural dimension of the extrudates, establishing how BT, feed moisture, SPC/MS ratio and MP level govern melt rheology and the physical architecture of the product (residence time, torque, specific mechanical energy, expansion index, apparent density, hardness, hydration indices, pH and total CO difference). Demonstrating that a formulation is technologically feasible, however, does not by itself establish that the resulting snack is nutritionally adequate, retains bioactive potential or is acceptable to consumers. The present study therefore addresses the complementary question of product quality from the nutritional, functional and sensory standpoint: How the same processing and formulation variables affect nutrient retention, antioxidant capacity, individual CO development and consumer acceptance of the extrudate. This distinction is not incidental, since conditions that maximise structural expansion or minimise density do not necessarily coincide with those that best preserve nutrients and bioactive compounds or that consumers prefer; capturing this trade‐off requires the nutritional, antioxidant and sensory dataset generated here, which has not been reported elsewhere for MP‐enriched soy protein–maize starch extrudates evaluated under the same experimental design as their corresponding process/structural characterisation.

## 2. Materials and Methods

### 2.1. Raw Materials

Melons used as the source of peel by‐product were obtained fresh from a local retail market located in San Juan Bautista Tuxtepec, Oaxaca, Mexico. The soy protein concentrate employed in the formulations was supplied by the commercial brand FTT (Fudtech, Food Technologies Trading S.A. de C.V., Mexico); on a dry basis, this ingredient contained 47.71/100 g carbohydrates, 37.92/100 g protein, 5.87/100 g ash and 0.34/100 g fat. Maize starch was supplied by IMSA (Industrializadora de Maíz S.A. de C.V., Tlalnepantla de Baz, State of Mexico, Mexico), with a dry‐basis composition of 87.23/100 g carbohydrates, 0.88/100 g protein, 0.20/100 g ash and 0.30/100 g fat. These proximate values for SPC and MS correspond to the same raw material batches and analyses reported in the companion study by Hernández‐Santos et al. [7] and are reproduced here for context rather than redetermined.

### 2.2. Preparation of MP Flour

Fruit washing and peel removal were performed according to the procedure reported by Téllez‐Morales et al. [8]. The recovered peels were cut into pieces of roughly 1 cm and dehydrated in a tray dryer at 60°C for 8 h. Once dried, the material was ground in a domestic mill (KRUPS, Model GX410011, Mexico) and passed through a sieve to reach a particle size of 0.59 mm (Mesh #30); this particle size was chosen because it favours a uniform blend with the starch–protein matrix and consistent behaviour during extrusion, whilst avoiding the excessive breakdown of the expandable structure that is typically caused by coarser fibre particles. The resulting MP flour contained 55.54/100 g carbohydrates, 21.05/100 g fibre, 8.76/100 g ash, 3.21/100 g protein and 1.27/100 g fat. These proximate values for the MP flour likewise correspond to the same raw material batch characterised in the companion study by Hernández‐Santos et al. [7] and are reproduced here for context rather than redetermined.

### 2.3. Preparation of the Blends

Blends were prepared by combining the raw materials in the proportions established by the experimental design (Table 1); every formulation variable expressed in grammes per 100 g refers to the share of each component within 100 g of the total dry mix, prior to moisture adjustment and extrusion. Distilled water was then added manually to bring each blend to its target moisture level, ensuring an even distribution throughout the mixture. The moisture‐adjusted samples were sealed in plastic bags and held at 4°C for 12 h so that water would distribute uniformly within the matrix before extrusion [9].

**Table 1:** Experimental data of extruded snack for response surface analysis.

Tr	Independent variables				Dependent variables																
	BT (°C)	MC (g/100 g)	SPC/MS (g/100 g)	MP (g/100 g)	Moisture (g/100 g)	Ash (g/100 g)	Protein (g/100 g)	Fat (g/100 g)	Fibre (g/100 g)	Carbohydrates (g/100 g)	*L* ^∗^	*a* ^∗^	*b* ^∗^	DPPH (%)	ABTS (%)	AR	CO	FL	TEX	AP	OA
1	132.5	18.25	28.75	13.75	9.09	3.02	12.83	0.16	6.49	67.98	67.39	3.13	21.17	15.02	39.79	5.97	5.70	5.70	6.83	6.23	6.43
2	157.5	18.25	28.75	13.75	8.97	2.71	12.39	1.30	7.94	67.70	67.14	3.41	23.14	15.49	39.96	5.97	5.70	6.07	7.27	6.17	6.80
3	132.5	22.75	28.75	13.75	9.20	3.01	13.41	0.57	6.18	66.89	67.06	1.65	22.68	12.34	39.86	5.43	4.77	5.07	4.77	5.23	5.33
4	157.5	22.75	28.75	13.75	8.91	3.46	12.66	0.50	9.58	67.54	68.27	1.90	22.55	12.95	41.87	5.73	5.47	5.67	5.57	5.83	6.10
5	132.5	18.25	36.25	13.75	9.24	3.34	15.31	0.49	6.61	64.70	66.39	4.52	22.41	18.01	60.54	6.60	6.63	7.07	7.10	7.03	7.40
6	157.5	18.25	36.25	13.75	8.61	3.53	15.00	0.41	6.91	65.53	67.93	3.99	23.12	26.11	59.60	6.07	6.17	6.60	6.60	6.40	6.70
7	132.5	22.75	36.25	13.75	8.85	3.32	14.98	0.46	7.52	65.46	67.17	2.44	22.58	16.57	44.57	5.33	5.37	5.57	5.63	5.77	5.80
8	157.5	22.75	36.25	13.75	8.72	2.99	15.58	0.20	3.00	65.58	66.96	2.56	22.48	15.09	47.36	5.50	5.47	5.67	5.80	5.80	5.80
9	132.5	18.25	28.75	21.25	8.46	3.30	11.65	0.30	9.69	65.58	68.45	3.20	22.91	26.28	52.84	5.93	5.20	5.87	6.03	5.43	6.40
10	157.5	18.25	28.75	21.25	9.03	3.45	10.63	0.28	10.85	65.91	67.35	2.32	22.91	24.87	46.33	5.77	5.43	6.10	5.80	5.77	6.23
11	132.5	22.75	28.75	21.25	8.64	3.40	10.78	0.25	10.7	66.22	70.17	1.53	23.50	20.71	39.53	5.37	4.83	4.77	4.47	5.23	5.20
12	157.5	22.75	28.75	21.25	8.82	3.32	12.82	0.32	9.97	64.01	69.15	1.19	23.40	19.59	39.12	5.73	5.30	5.30	4.93	5.37	5.53
13	132.5	18.25	36.25	21.25	8.32	3.92	14.55	0.36	8.83	62.15	64.98	3.50	22.46	25.05	51.91	5.80	6.10	6.43	6.87	6.53	7.07
14	157.5	18.25	36.25	21.25	8.36	3.89	12.67	3.25	8.67	61.12	65.67	3.25	22.43	26.01	44.74	5.93	5.67	6.20	5.73	5.80	6.37
15	132.5	22.75	36.25	21.25	8.77	3.71	10.64	0.46	10.65	65.71	67.75	1.78	24.15	19.19	36.67	5.73	4.83	5.53	4.80	5.10	5.60
16	157.5	22.75	36.25	21.25	8.63	3.80	13.69	0.67	9.80	62.51	69.37	0.98	23.43	19.31	38.77	5.53	5.60	5.77	6.23	6.23	6.23
17	120.0	20.50	32.50	17.50	8.76	3.17	15.59	0.47	7.79	63.19	70.48	1.25	23.52	17.14	37.82	5.73	6.10	6.27	6.10	6.23	6.40
18	170.0	20.50	32.50	17.50	8.75	3.12	14.56	0.21	7.50	64.54	68.61	2.02	23.40	17.29	34.81	5.87	5.50	6.13	6.27	5.70	6.33
19	145.0	16.00	32.50	17.50	8.57	3.11	13.25	1.26	8.68	64.99	65.87	4.93	21.36	22.00	34.84	6.40	6.63	7.17	7.53	6.93	7.23
20	145.0	25.00	32.50	17.50	9.15	3.11	13.12	0.39	4.45	65.43	70.12	1.01	23.66	17.45	37.37	5.83	5.40	5.50	5.20	5.73	5.67
21	145.0	20.50	25.00	17.50	8.73	2.64	8.75	0.23	8.81	70.84	72.04	1.11	23.43	19.59	36.18	5.07	5.50	6.00	5.83	5.73	6.10
22	145.0	20.50	40.00	17.50	8.79	3.71	15.31	0.16	7.77	63.22	68.02	2.43	22.55	17.58	44.63	5.73	5.80	5.70	5.17	5.87	5.87
23	145.0	20.50	32.50	10.00	9.13	2.72	14.26	0.33	2.42	68.52	71.71	2.99	22.43	13.34	31.91	5.93	6.30	6.83	6.67	6.63	6.90
24	145.0	20.50	32.50	25.00	8.37	3.76	11.65	0.19	10.59	63.44	67.71	1.72	23.32	18.29	35.94	5.67	4.97	6.13	5.37	5.03	5.73
25	145.0	20.50	32.50	17.50	8.74	3.27	15.75	0.18	8.11	63.24	68.74	2.47	23.30	17.89	38.27	5.87	5.93	6.10	6.47	5.77	6.10
26	145.0	20.50	32.50	17.50	8.90	3.02	15.04	0.15	9.23	64.07	70.57	2.43	23.56	17.44	33.55	5.93	5.67	6.27	6.03	6.03	6.33
27	145.0	20.50	32.50	17.50	8.83	3.21	15.46	0.12	7.94	63.56	71.03	1.93	24.00	17.56	38.75	5.87	5.93	6.33	6.40	6.10	6.50
28	145.0	20.50	32.50	17.50	8.88	3.29	14.58	0.14	7.33	64.30	68.53	2.37	22.66	19.24	36.67	6.13	5.83	6.47	6.37	6.20	6.53
29	145.0	20.50	32.50	17.50	8.77	2.94	13.10	0.61	9.56	65.77	70.15	1.94	22.90	18.55	42.15	5.97	6.13	6.53	6.40	6.20	6.63
30	145.0	20.50	32.50	17.50	8.56	3.20	13.84	0.10	8.82	65.49	70.63	1.84	23.35	17.63	40.69	6.00	6.10	6.40	6.03	6.30	6.40

*Note:* The independent variables BT, MC, SPC/MS and MP (Treatments 1–30) correspond to the same central composite design and extrusion trials reported in the companion study by Hernández‐Santos et al. [7] and are reproduced here for reference; they are not presented as a new result. All other columns (extrudate proximate composition, *L* ^∗^, *a* ^∗^, *b* ^∗^, DPPH, ABTS and sensory scores) are original data generated for the present study.

Abbreviations: AP, appearance; AR, aroma; BT, barrel temperature; CO, colour; FL, flavour; MC, moisture content; MP, melon peel; MS, maize starch; OA, overall acceptability; SPC, soy protein concentrate; TEX, texture; Tr, treatment.

### 2.4. Extrusion Process

Extrusion trials were performed on a laboratory‐scale single‐screw extruder (Extruder 19/25DN, Model 832005.007, Brabender GmbH & Co. KG, Germany) fitted with a 428 mm barrel, a 19 mm screw diameter and a 3: 1 compression ratio; a circular die of 4 mm internal diameter was used at the outlet. Temperatures along the barrel were fixed at 50°C (Zone 1), 80°C (Zone 2) and 100°C (Zone 3), whereas Zone 4 (die section) was varied between 120°C and 170°C according to the experimental design (Table 1). Throughout all runs, the material was fed at a constant rate of 60 g/min, and the screw was operated at a fixed speed of 150 rpm.

### 2.5. Characterisation of the Extruded Product

#### 2.5.1. Proximate Chemical Analysis

Proximate composition of the extrudates was assessed following the official AOAC procedures [10] for moisture (925.10), ash (923.03), protein (920.87), fat (920.39) and crude fibre (962.09). Carbohydrate content, in turn, was obtained by difference, subtracting the sum of moisture, protein, fat, ash and crude fibre (all in grammes per 100 g) from 100. Each determination was carried out in triplicate.

#### 2.5.2. CO Parameter Evaluation

Surface CO of the extrudates was measured with a HunterLab tristimulus colourimeter (MiniScan HunterLab, Model 45/0L, Hunter Associates Laboratory Inc., Reston, Virginia, United States), recording the parameters *L* ^∗^ (lightness), *a* ^∗^ (red–green chromaticity) and *b* ^∗^ (yellow–blue chromaticity). These *L* ^∗^, *a* ^∗^ and *b* ^∗^ readings correspond to the same colourimetric measurements used to calculate the total CO difference (*Δ* *E*) reported in the companion study by Hernández‐Santos et al. [7]; here, the individual coordinates are reported and discussed separately to relate specific browning mechanisms (Maillard reaction and phenolic oxidation) to the sensory CO and appearance (AP) scores collected in Section 2.5.4.

#### 2.5.3. Determination of Antioxidant Activity (2,2‐Diphenyl‐1‐Picrylhydrazyl [DPPH] and ABTS)

Before running the antioxidant assays, ground extrudate samples were extracted with methanol, and the suspension was centrifuged to recover the supernatant used in both the DPPH and ABTS determinations. Radical‐scavenging activity against DPPH• was evaluated from a 0.1 mM methanolic stock solution; a calibration curve was built using 0.02, 0.04, 0.06, 0.08 and 0.1 mM dilutions, with methanol as the blank. For the assay, 50 *μ*L of the methanolic extract (100 ppm) was added to 2.0 mL of the DPPH solution (0.1 mM), and absorbance was read at 517 nm, following the procedure described by Rodríguez‐Miranda et al. [11].

Antioxidant capacity was further estimated through the ABTS•^+^ radical cation decolourisation assay, based on the protocol of Re et al. [12]. The ABTS reagent (7 mM) was dissolved in distilled water and reacted with potassium persulphate (2.45 mM) to generate the ABTS•^+^ radical, which was left to develop in the dark at room temperature for 12–16 h. Before use, this solution was diluted with ethanol (pH 7.4) until reaching an absorbance of 0.70 ± 0.02 at 734 nm and was then equilibrated at 30°C. The assay itself involved combining 10 *μ*L of the methanolic extract with 990 *μ*L of the diluted ABTS•^+^ solution and reading the absorbance at 734 nm.

#### 2.5.4. Sensory Analysis

A 9‐point hedonic scale (1 = *dislike extremely*; 9 = *like extremely*) was used for sensory evaluation, applying the methodology of Ramírez‐Rivera et al. [13]. One hundred untrained panellists, drawn from amongst students and staff of the Instituto Tecnológico de Tuxtepec, took part; their ages ranged from 19 to 50 years. Before tasting, every participant was briefed on the purpose of the study and the ingredients used, and each signed an informed consent form.

Testing took place over four sessions in the institute′s sensory laboratory under white fluorescent lighting, with samples presented to each panellist in randomised order. Panellists scored aroma (AR), CO, flavour (FL), TEX, AP and overall acceptability (OA) for each sample, after receiving a short briefing on how to apply the 9‐point scale to each attribute. Samples were tasted one at a time in random sequence, and panellists rinsed with water between samples to limit carry‐over effects on perception.

#### 2.5.5. Experimental Design and Data Analysis

The experiments followed a central composite design under the RSM framework (Table 1), processed with Design‐Expert software Version 7.0.0 (Stat‐Ease Inc., Minneapolis, Minnesota, United States). BT (120°C–170°C), feed MC (16–25/100 g), the SPC/MS (25–40/100 g) ratio and MP (10–25/100 g) level served as independent variables, whereas moisture, ash, fat, protein, fibre and carbohydrate contents, the CO coordinates *L* ^∗^, *a* ^∗^ and *b* ^∗^, DPPH and ABTS inhibition and the sensory scores (AR, CO, FL, TEX, AP and OA) were treated as response variables. Multiple linear regression was used to fit the experimental data, and the significance of each model term was tested by analysis of variance (ANOVA) for every response. This manuscript and the companion paper by Hernández‐Santos et al. [7] were generated from the same 30‐treatment extrusion campaign under the design described above; the two manuscripts report complementary, nonoverlapping response variables: process and technofunctional/structural parameters in [7] and nutritional composition, CO coordinates, antioxidant activity and sensory quality in the present study.

## 3. Results and Discussion

### 3.1. Effect of BT, MC, SPC/MS Ratio and MP Content on Chemical Composition

Moisture values across the extruded snacks spanned 8.32–9.24/100 g (Table 1), with Treatment 5 (BT = 132.5^°^C, MC = 18.25/100 g, SPC/MS = 36.25/100 g and MP = 13.75/100 g) giving the highest reading and Treatment 13 (BT = 132.5^°^C, MC = 18.25/100 g, SPC/MS = 36.25/100 g and MP = 21.25/100 g) the lowest. Because BT, MC and SPC/MS were identical between these two treatments, the drop associated with raising MP content points to MP as the dominant driver of this response. A quadratic model captured 75% of the variability observed (*R* ^2^ = 0.75; Table 2) and reached significance, with a nonsignificant lack of fit confirming it described the data adequately across the tested range. Amongst the model terms, only the linear MP effect (*D*) and the BT × MP interaction (AD) reached significance; BT, MC and SPC/MS did not contribute significant linear effects on their own.

**Table 2:** Analysis of variance of the independent and dependent variables of extruded snacks.

Source	DF	Mean squares																
		Moisture	Ash	Protein	Fat	Fibre	Carbohydrates	*L* ^∗^	*a* ^∗^	*b* ^∗^	DPPH	ABTS	AR	CO	FL	TEX	AP	OA
Model	14	0.037^a^	0.200^a^	0.111^a^	1.55	3.74^a^	0.415^a^	0.211	2.690^a^	0.106^a^	0.316^a^	0.299	0.007^a^	0.018^a^	0.020^a^	0.045^a^	0.017^a^	0.021^a^
A‐BT	1	0.000	0.000	0.002	0.075	0.003	0.001	0.000	0.001	0.001	0.021	0.043	0.000	0.000	0.003	0.007	0.000	0.001
B‐MC	1	0.003	0.000	0.005	0.201	0.118	0.003	0.056^a^	1.860^a^	0.036^a^	1.300^a^	0.848	0.041^a^	0.101^a^	0.175^a^	0.376^a^	0.089^a^	0.199^a^
C‐SPC/MS	1	0.003	0.079^a^	0.663^a^	0.047	0.194	0.186^a^	0.043^a^	0.235^a^	0.000	0.122	0.857	0.007^a^	0.030^a^	0.024	0.006	0.023^a^	0.010
D‐MP	1	0.020^a^	0.096^a^	0.322^a^	0.004	2.61^a^	0.128^a^	0.002	0.317^a^	0.022^a^	2.030^a^	0.041	0.003	0.045^a^	0.013	0.093^a^	0.068^a^	0.027^a^
AB	1	0.000	0.000	0.093^a^	0.235	0.08	0.005	0.000	0.000	0.009	0.075	0.146	0.004	0.021^a^	0.007	0.052^a^	0.024^a^	0.022^a^
AC	1	0.003	0.000	0.004	0.006	0.265	0.001	0.005	0.004	0.003	0.051	0.001	0.002	0.006	0.012	0.006	0.003	0.010
AD	1	0.006^a^	0.000	0.013	0.059	0.000	0.014	0.001	0.052	0.008	0.060	0.083	0.000	0.001	0.000	0.002	0.002	0.000
BC	1	0.000	0.012	0.029	0.067	0.015	0.017^a^	0.001	0.009	0.000	0.049	0.296	0.002	0.007	0.001	0.017	0.002	0.000
BD	1	0.002	0.001	0.009	0.024	0.064	0.004	0.020	0.011	0.007	0.009	0.115	0.006^a^	0.004	0.000	0.008	0.006	0.001
CD	1	0.000	0.003	0.014	0.329^a^	0.035	0.001	0.008	0.026	0.001	0.422^a^	1.040	0.000	0.001	0.000	0.007	0.000	0.002
*A* ^2^	1	0.000	0.003	0.003	0.080	0.000	0.003	0.015	0.017	0.000	0.004	0.127	0.002	0.007	0.012	0.002	0.003	0.001
*B* ^2^	1	0.000	0.001	0.074	0.456^a^	0.099	0.003	0.061^a^	0.101^a^	0.015	0.155	0.112	0.002	0.001	0.006	0.000	0.001	0.000
*C* ^2^	1	0.000	0.003	0.279^a^	0.012	0.021	0.038^a^	0.007	0.007	0.003	0.054	0.607	0.024^a^	0.016	0.040^a^	0.055^a^	0.010	0.017^a^
*D* ^2^	1	0.000	0.006	0.102^a^	0.040	0.226	0.013	0.012	0.039	0.005	0.048	0.008	0.002	0.018^a^	0.001	0.009	0.010	0.002
Residual	15	0.012	0.057	0.018	1.040	1.26	0.054	0.138	0.305	0.037	0.065	0.252	0.001	0.004	0.005	0.006	0.004	0.003
Lack of fit	10	0.010	0.049	0.018	0.895	1.15	0.033	0.118	0.257	0.025	0.094	0.348	0.002	0.005	0.008	0.008	0.006	0.004
Pure error	5	0.002	0.008	0.018	0.150	0.106	0.020	0.020	0.049	0.012	0.007	0.061	0.000	0.001	0.000	0.001	0.002	0.001
*R* ^2^		0.75	0.78	0.85	0.60	0.75	0.89	0.60	0.90	0.74	0.82	0.53	0.83	0.81	0.78	0.88	0.79	0.86

Abbreviations: AP, appearance; AR, aroma; BT, barrel temperature; CO, colour; DF, degrees of freedom; FL, flavour; MC, moisture content; MP, melon peel; MS, maize starch; OA, overall acceptability; SPC, soy protein concentrate; TEX, texture.

^a^Significance at *p* < 0.05.

**Figure 1:**
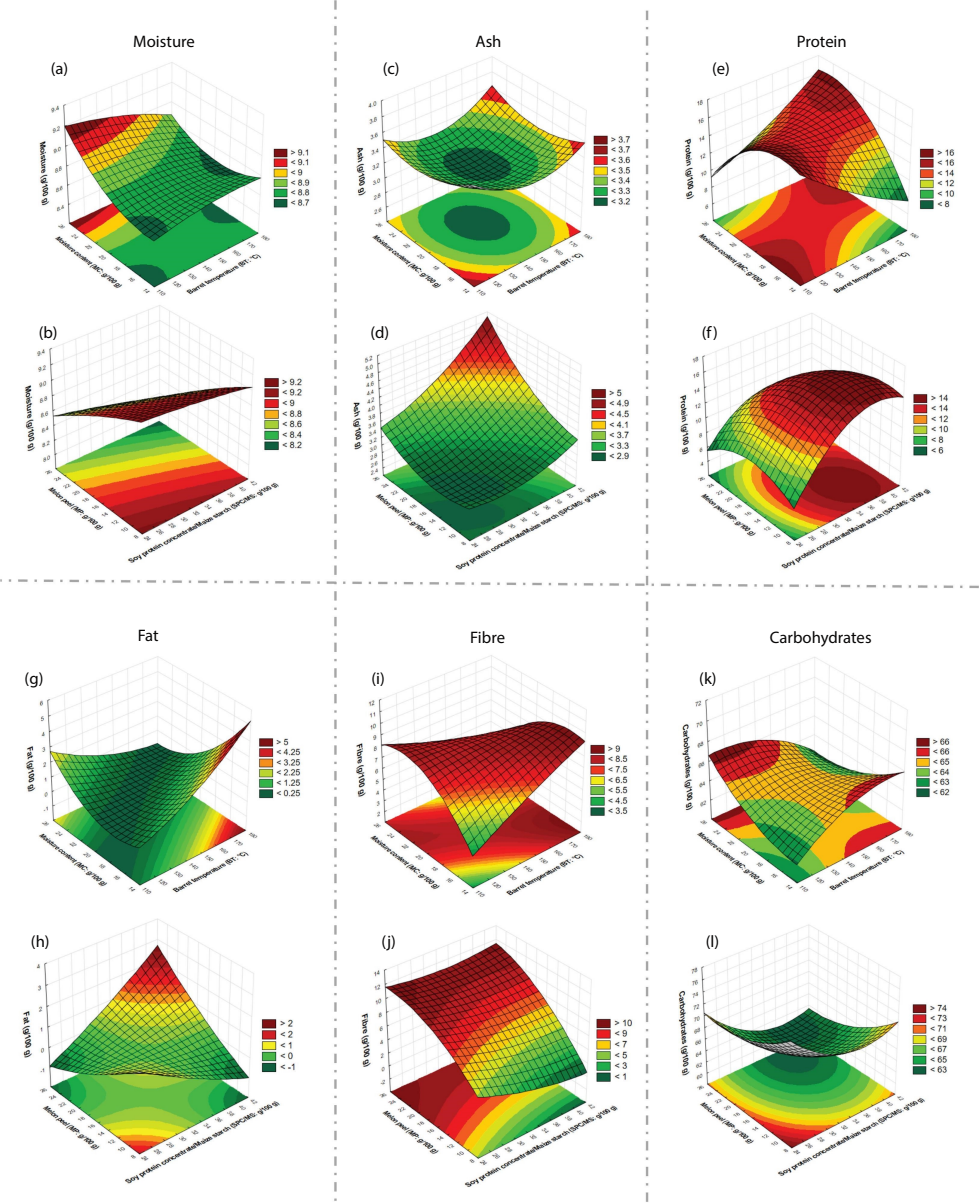
Response surface plots showing the effects of barrel temperature (BT), moisture content (MC), melon peel (MP) and soy protein concentrate/maize starch (SPC/MS) ratio on (a,b) moisture, (c,d) ash, (e,f) protein, (g,h) fat, (i,j) fibre and (k,l) carbohydrates.

This is borne out by the estimated coefficients (Table 3): MP carried a significant negative linear coefficient (*b*_4_ = −0.029; *p* < 0.05), whereas its interaction with BT was positive and significant (*b*_14_ = 0.019; *p* < 0.05), together pointing to a moisture‐lowering effect of MP that eases somewhat as extrusion temperature rises. The response surfaces in Figure 1a,b agree with this reading: The BT–MC plane shows only mild variation, whereas the SPC/MS–MP plane shows a steeper gradient tied to MP content. A comparable pattern, fibre‐rich formulations yielding drier, crispier extrudates, has been reported elsewhere for plant by‐product‐enriched extrudates [5, 14, 15].

**Table 3:** Regression coefficients from a second‐order model of relationships between response and independent variables for extruded snack.

Coe	Moisture	Ash	Protein	Fat	Fibre	Carbohydrates	*L* ^∗^	*a* ^∗^	*b* ^∗^	DPPH	ABTS	AR	CO	FL	TEX	AP	OA
*b*_0_	2.96^a^	1.78^a^	3.82^a^	0.437	2.91^a^	8.03^a^	8.36	1.47^a^	4.83^a^	4.25^a^	6.19	2.44^a^	2.44^a^	2.52^a^	2.51^a^	2.47^a^	2.53^a^
*b*_1_	−0.004	0.000	−0.003	0.056	−0.011	−0.005	−0.003	−0.006	0.006	0.030	−0.043	0.003	0.003	0.011	0.017	−0.001	0.005
*b*_2_	0.011	−0.001	−0.005	−0.092	−0.070	0.011	0.048^a^	−0.278^a^	0.039^a^	−0.233^a^	−0.188	−0.041^a^	−0.065^a^	−0.085^a^	−0.125^a^	−0.061^a^	−0.091^a^
*b*_3_	−0.011	0.057^a^	0.166^a^	0.044	−0.090	−0.088^a^	−0.042^a^	0.099^a^	−0.004	0.071	0.189	0.017^a^	0.035^a^	0.032	0.016	0.031^a^	0.021
*b*_4_	−0.029^a^	0.063^a^	−0.116^a^	0.013	0.330^a^	−0.073^a^	−0.009	−0.115^a^	0.030^a^	0.291^a^	−0.041	−0.011	−0.044^a^	−0.024	−0.062^a^	−0.053^a^	−0.034^a^
*b*_12_	−0.003	0.003	0.076^a^	−0.121	−0.071	−0.017	0.003	0.002	−0.024	−0.068	0.096	0.016	0.036^a^	0.021	0.057^a^	0.038a	0.037^a^
*b*_13_	−0.013	−0.005	0.015	0.019	−0.129	−0.007	0.018	−0.016	−0.012	0.057	0.008	−0.012	−0.019	−0.027	−0.019	−0.015	−0.026
*b*_14_	0.019^a^	0.003	0.029	0.061	−0.001	−0.029	−0.008	−0.057	−0.022	−0.061	−0.072	0.002	0.009	0.002	−0.004	0.012	−0.004
*b*_23_	0.005	−0.027	−0.043	−0.065	−0.030	0.032^a^	0.008	−0.024	0.001	−0.056	−0.136	−0.012	−0.020	−0.008	0.032	−0.011	−0.003
*b*_24_	0.010	−0.009	−0.023	−0.039	0.063	0.016	0.036	−0.026	0.021	−0.023	−0.085	0.021	0.016	0.002	0.023	0.021	0.009
*b*_34_	−0.002	0.013	−0.030	0.143^a^	0.047	−0.006	−0.023	−0.040	−0.009	−0.162^a^	−0.256	−0.002	−0.007	−0.005	0.021	0.005	0.012
*b*_11_	−0.001	0.010	0.011	0.054	−0.001	−0.011	−0.023	−0.025	0.002	0.004	0.068	−0.008	−0.016	−0.021	−0.009	−0.011	−0.006
*b*_22_	0.004	0.007	−0.052	0.129^a^	−0.060	0.010	−0.047^a^	0.061^a^	−0.023	0.075	0.064	0.008	−0.006	−0.015	−0.003	0.007	−0.003
*b*_33_	−0.001	0.010	−0.101^a^	0.021	0.028	0.037^a^	−0.016	−0.016	−0.010	0.044	0.149	−0.031^a^	−0.024	−0.038^a^	−0.045^a^	−0.019	−0.025^a^
*b*_44_	−0.001	0.015	−0.061^a^	0.038	−0.091	0.022	−0.021	0.038	−0.013	−0.042	0.017	−0.008	−0.026^a^	−0.007	−0.018	−0.019	−0.009

Abbreviations: AP, appearance; AR, aroma; CO, colour; Coe, coefficients; FL, flavour; OA, overall acceptability; TEX, texture.

^a^Significance at *p* < 0.05.

MP flour is itself hydrophilic and can absorb water, yet within the extruder, its fibre fraction competes with starch and protein for the water present in the mix. That competition leaves less free water for gelatinising starch and plasticising the melt, which pushes up local melt viscosity and encourages more flashing‐off of steam once the material exits the die, so the extrudate can end up drier than the fibre′s hydrophilicity alone would predict. Insoluble fibre particles add a further complication: They can break up the continuity of the viscoelastic starch network, weakening its ability to hold water inside the expanded cells and letting more moisture escape as the product expands and cools.

The ash content of the extrudates ranged from 2.64 to 3.92/100 g (Table 1), a spread that points to both formulation and processing conditions playing a role. The lowest reading (2.64/100 g) came from Treatment 21 (BT = 145^°^C, MC = 20.5/100 g, SPC/MS = 25/100 g and MP = 17.5/100 g), and the highest (3.92 g/100 g) came from Treatment 13 (BT = 132.5^°^C, MC = 18.25/100 g, SPC/MS = 36.25/100 g and MP = 21.25/100 g). Higher MP and SPC/MS levels went hand in hand with a richer mineral fraction in the product, which tracks with what these two ingredients themselves contribute in minerals.

A quadratic model fit the ash data significantly (*p* < 0.05; Table 2), with *R* ^2^ above 0.80 and a nonsignificant lack of fit—together indicating the model captured the experimental variability well. MP content (*D*) and the SPC/MS ratio (*C*) stood out as the dominant terms, along with a handful of second‐order interactions, a pattern also visible in Table 3 coefficients.

In Figure 1c (BT–MC), the surface curves gently, with ash staying moderate at intermediate temperature and moisture and rising slightly only at extreme combinations. Figure 1d, by contrast, shows a steep climb in ash as both SPC/MS and MP rise, underscoring that formulation, rather than thermal processing, drives this response. Other work on plant by‐product‐enriched extrudates rich in fibre and minerals reports the same pattern incorporation of plant matrices pushing ash content upward [5, 15, 16], reinforcing that MP is a substantial contributor to mineral enrichment here.

Protein content tracked the experimental design as expected given the compositional nature of the system, spanning roughly 8.75–15.75/100 g (Table 1) in line with what is typical for starch–protein matrices carrying SPC. A quadratic model fit these data with high significance (*p* < 0.05) and *R* ^2^ above 0.95 (Table 2). Table 3′s effect analysis singled out the linear SPC/MS term (*C*) as the main driver (*p* < 0.05); BT (*A*) and MC (*B*) contributed no significant linear effects, though small quadratic terms and interactions with MP were present.

Table 3′s regression coefficients confirm this: SPC/MS carried a significant positive coefficient (*b*_3_ > 0; *p* < 0.05), meaning more protein concentrate in the mix translated directly into more protein in the extrudate, unsurprising from a formulation standpoint. BT and MC, on the other hand, were either nonsignificant or contributed little, suggesting that within the range tested, the extrusion step itself did not strip out meaningful amounts of protein. Formulation, rather than heat‐driven degradation, was the main lever governing protein content.

Figure 1e (protein vs. BT–MC) is essentially flat, without pronounced gradients consistent with the lack of significant linear temperature or moisture effects on total protein. This matches what the literature generally reports: Extrusion drives structural denaturation, but conventional protein assays (Kjeldahl/Dumas) usually do not register a real loss of total protein unless degradation is extreme [17].

Figure 1f (protein vs. SPC/MS–MP), on the other hand, climbs clearly along the SPC/MS axis; more protein in the formulation means more protein in the product. That protein content held steady across BT and MC likely reflects extrusion′s tendency to reshape proteins conformationally (denaturing, aggregating them) without actually removing nitrogen. Work on protein–starch extrudates backs this up: Extrusion can alter the secondary and tertiary structure of proteins, whereas total measured protein stays put, even as functional traits like solubility and digestibility shift [18, 19].

In systems built around plant proteins, rising temperature can even encourage protein–starch interactions via hydrophobic bonding and disulphide links, reshaping TEX and microstructure without changing the overall protein share [20].

Beyond simply raising protein content, a higher SPC/MS ratio also reshapes melt rheology because it necessarily comes at the expense of maize starch. Starch is what drives gelatinisation, bubble growth and expansion during extrusion, so scaling it back can hinder the formation of a continuous viscoelastic network able to hold vapour pressure until the die exit. Soy protein behaves differently in the melt; it tends to add elasticity and favour denser, more compact cell structures. Protein denaturation and protein–starch interactions occurring during processing can also affect bubble stability, cell wall formation and final TEX, which is why shifting the SPC/MS ratio touches not just nutritional composition but expansion, TEX and overall structure as well.

MP itself had no significant linear effect on protein content (Table 2), unsurprising given its comparatively low protein contribution relative to SPC, though its interaction with SPC/MS may still shape final protein levels through simple compositional dilution. This lines up with findings for other agroindustrial by‐product‐enriched extrudates, where protein content tracks the amount of protein ingredient added almost exclusively, rather than any thermal effect [21].

The fat content of the extruded snacks ranged approximately from 0.10 to 3.25/100 g (Table 1), pointing to a joint influence of formulation and processing on this response. The minimum (0.10/100 g) came from Treatment 30 (BT = 145^°^C, MC = 20.5/100 g, SPC/MS = 32.5/100 g and MP = 17.5/100 g), and the maximum (3.25/100 g) came from Treatment 14 (BT = 157.5^°^C, MC = 18.25/100 g, SPC/MS = 36.25/100 g and MP = 21.25/100 g). Higher BT paired with lower feed moisture appears to favour higher apparent fat readings, which could reflect how the extruded matrix′s structure shifts in ways that affect whether lipids are retained or released during processing.

The quadratic fit for fat was only moderate (*R* ^2^ = 0.60; Table 2), and overall model significance was limited across the tested range, though a nonsignificant lack of fit (*p* > 0.05) still supports its suitability for describing this response. Table 3′s coefficients point to MP content and the SPC/MS ratio as the strongest drivers, alongside a few process–formulation interactions.

Figure 1g (BT–MC) shows fat rising at higher temperature and lower moisture, which could reflect greater thermal degradation of the matrix or shifts in how lipids distribute during processing. Figure 1h shows fat climbing progressively as both SPC/MS and MP increase, confirming that formulation also plays a part here. This echoes what has been reported for other plant by‐product‐enriched extruded snacks, where fibre‐rich, structurally complex ingredients alter lipid–starch interactions during extrusion [5, 15, 16].

Fibre content varied widely across treatments (2.42–10.85/100 g, Table 1), and within this domain, formulation clearly outweighed the purely thermomechanical variables in shaping the final fibre fraction. The peak (10.85/100 g) came under high MP (21.25/100 g) combined with fairly low MC (18.25/100 g), whereas the trough (2.42/100 g) appeared when MP sat at its minimum (10.00/100 g) (Table 1). This dilution/enrichment pattern tracks the raw material′s own composition directly: Since MP is fundamentally a fibre‐rich ingredient, adding more of it simply raises the fibre content of the finished product.

The RSM analysis backs this up statistically. Fibre content fit a significant quadratic model (Table 3), and amongst the ANOVA terms, only D‐MP reached significance (*p* < 0.05); BT, MC and SPC/MS along with their interactions and quadratic terms, contributed nothing significant (Table 2). As per Table 3, MP′s linear coefficient was positive and significant (*b*_4_ = +0.330; *p* < 0.05), whereas BT, MC and SPC/MS coefficients were small and nonsignificant, meaning total fibre in this system was essentially a function of how much MP was loaded, staying largely unaffected by extrusion conditions across the ranges tested.

The response surfaces reinforce this reading. Figure 1i (fibre vs. BT–MC) is nearly flat, with only minor noise‐level variation, showing that neither BT nor MC meaningfully changed fibre content once MP was held fixed. Figure 1j (fibre vs. SPC/MS–MP), meanwhile, climbs sharply along the MP axis; fibre rises markedly as the peel fraction grows, whereas SPC/MS barely registers, matching its nonsignificant status in the model (Tables 2 and 3).

This lines up with the broader literature on fibre‐rich by‐products in extrusion, where the type and dose of fibre added are typically what sets the final fibre content and its knock‐on effects on structure, expansion and TEX, whereas processing conditions mainly shift the soluble/insoluble split (WAI/WSI) rather than the total measured by proximate analysis [21]. Work examining fibre source, dose and processing conditions together similarly finds that although extrusion can reshape polysaccharide microstructure and how dietary fibre distributes, the ingredient dosage remains the chief determinant of the reported fibre ‘content’ [22].

MP′s dominance in setting fibre content matters practically: More fibre usually means denser matrices and less expansion, but also greater nutritional and functional value. In practice, this means fibre targets can be dialled in mainly through MP dosing, leaving BT and MC free to fine‐tune structural and functional attributes without disturbing the fibre level much. This also fits with broader trends towards valorising *Cucumis melo* by‐products as fibre sources within extruded foods more generally [23, 24].

Carbohydrate levels in the extruded snacks ranged from 61.12 to 70.84/100 g (Table 1), mirroring how formulation and processing jointly shape the matrix′s dominant starchy fraction. The floor (61.12/100 g) belonged to Treatment 14 (BT = 157.5^°^C, MC = 18.25/100 g, SPC/MS = 36.25/100 g and MP = 21.25/100 g), and the ceiling (70.84/100 g) belonged to Treatment 21 (BT = 145^°^C, MC = 20.5/100 g, SPC/MS = 25/100 g and MP = 17.5/100 g). This largely comes down to dilution: As nonstarchy ingredients like SPC and MP take up a bigger share of the mix, the starchy fraction shrinks correspondingly, whereas protein and fibre climb.

Carbohydrates fit a significant quadratic model (*p* < 0.05; Table 2) with a solid *R* ^2^ of 0.88, and a nonsignificant lack of fit backs its validity across the domain tested. The SPC/MS ratio (*C*) and MP content (*D*) emerged from Table 3 as the strongest independent drivers, joined by a few quadratic formulation‐related terms.

Figure 1k (BT–MC) shows a mild decline in carbohydrate content as temperature and moisture rise, likely tied to starch′s structural changes during gelatinisation and extrusion‐driven dextrinisation. Figure 1l shows carbohydrates steadily falling as SPC/MS and MP both increase, reflecting starch being partly displaced by protein and fibre. This mirrors what is well documented for extruded snacks carrying plant by‐products or protein concentrates, where formulation shifts noticeably reshape the carbohydrate share of the finished product [5, 15, 16].

### 3.2. Effect of BT, MC, SPC/MS Ratio and MP Content on CO Parameters (*L* ^∗^, *a* ^∗^ and *b* ^∗^)

Lightness (*L* ^∗^) of the extrudates spanned 64.98 to 72.04 (Table 1), a fairly wide range reflecting how much processing and formulation together shift the product′s brightness. The dimmest reading (64.98) belonged to Treatment 13 (BT = 132.5^°^C, MC = 18.25/100 g, SPC/MS = 36.25/100 g and MP = 21.25/100 g), and the brightest (72.04) belonged to Treatment 21 (BT = 145^°^C, MC = 20.5/100 g, SPC/MS = 25/100 g and MP = 17.5/100 g). Both raising extrusion temperature and adding more nonstarchy ingredients appear to darken the extrudate, lowering its lightness.

**Figure 2:**
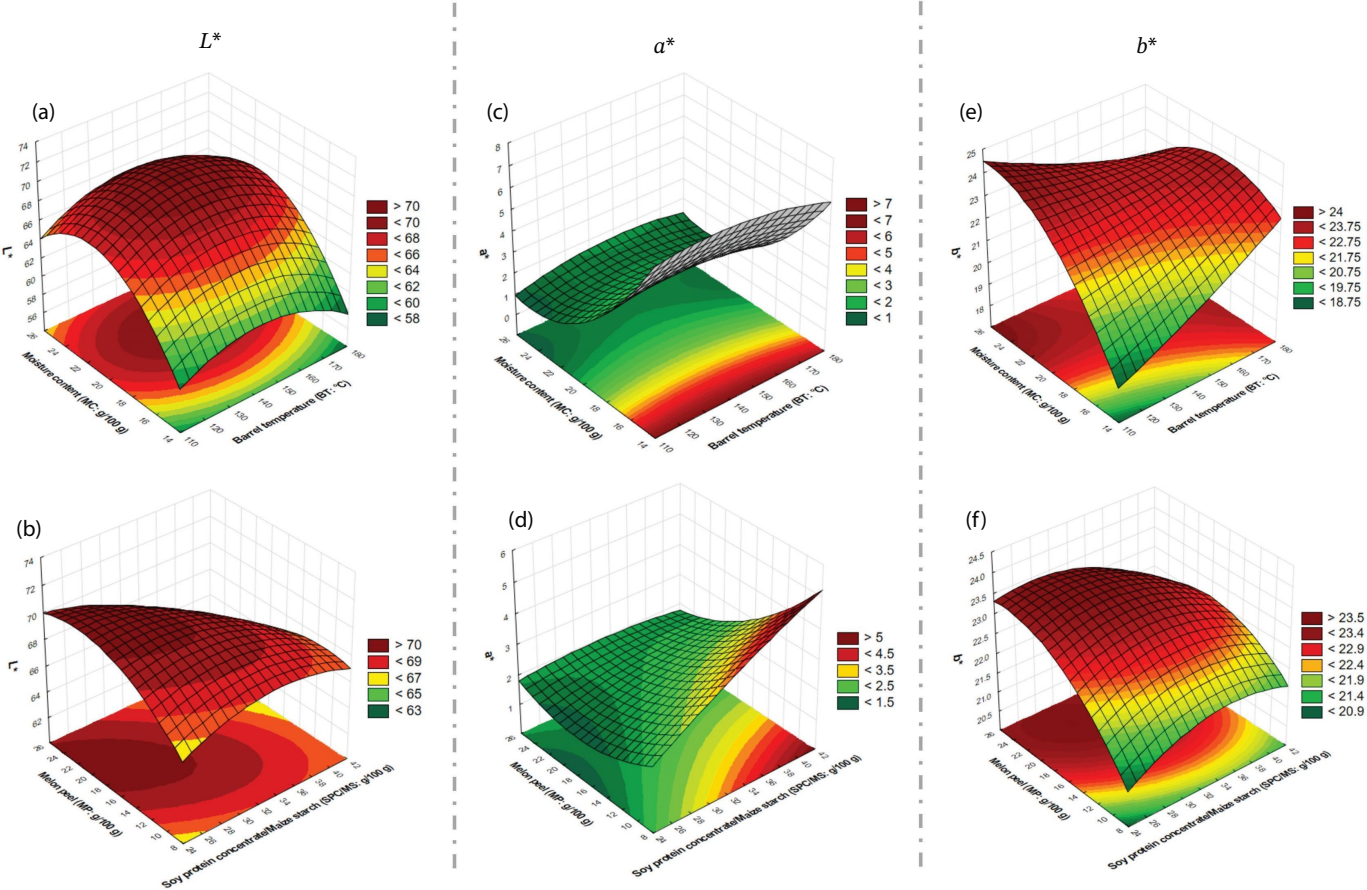
Response surface plots showing the effects of barrel temperature (BT), moisture content (MC), melon peel (MP) and soy protein concentrate/maize starch (SPC/MS) ratio on (a,b) *L* ^∗^, (c,d) *a* ^∗^ and (e,f) *b* ^∗^.

The quadratic model for *L* ^∗^ reached an acceptable *R* ^2^ of roughly 0.60 (Table 2); a moderate but workable fit across the domain tested and a nonsignificant lack of fit further supports it. Table 3 points to BT, the SPC/MS ratio and MP content as the main drivers, plus a few quadratic formulation terms. Figure 2a (BT–MC) shows *L* ^∗^ steadily dropping as BT climbs, an effect most visible at low moisture, plausibly tied to the same thermal browning discussed above for *L* ^∗^, including Maillard chemistry and pigment buildup during extrusion. Figure 2b shows lightness falling as SPC/MS and MP both rise, suggesting protein‐rich ingredients and phenolic compounds push the product towards darker tones. This is a well‐documented pattern in by‐product‐enriched extruded snacks: Higher processing temperature paired with more reactive compounds drives nonenzymatic browning, chiefly Maillard reactions and caramelisation, generating dark pigments and melanoidins that noticeably shift the product′s CO [1, 2, 5].

The red–green coordinate *a* ^∗^ covered 0.98–4.93 across the snacks (Table 1), bottoming out at Treatment 16 $\begin{array}{c} \left(\text{BT}=15722.7510036.25100.5^{{}^{\circ}}\text{C},\text{MC}=/\;\text{g},\text{SPC}/\text{MS}=/\;\text{g}\right. \end{array}$ and MP = 21.25/100 g) and peaking at Treatment 19 (BT = 145^°^C, MC = 16/100 g, SPC/MS = 32.5/100 g and MP = 17.5/100 g). Higher extrusion temperature combined with larger SPC and MP shares appears to favour redder tones. The *a* ^∗^ quadratic model was significant (*p* < 0.05) with *R* ^2^ of 0.90 (Table 2), and its lack of fit was nonsignificant (*p* > 0.05). Table 3′s coefficients again single out BT, the SPC/MS ratio and MP content as the most influential terms, plus some quadratic formulation contributions.

Figure 2c (BT–MC) shows *a* ^∗^ climbing with temperature, an effect sharper at lower moisture likely tied to intensifying Maillard chemistry during extrusion. Figure 2d shows *a* ^∗^ rising steadily with SPC/MS and MP, again pointing to protein‐rich ingredients and phenolic compounds feeding into browning pigment formation. Comparable trends turn up in other plant by‐product‐enriched extruded snacks, where higher temperature and protein content together generate coloured compounds and melanoidins that reshape the product′s CO [1, 2, 5].

The yellow–blue coordinate *b* ^∗^ ranged from 21.17 to 24.15 across treatments (Table 1). The low end (21.17) belonged to Treatment 1 (BT = 132.5^°^C, MC = 18.25/100 g, SPC/MS = 28.75/100 g and MP = 13.75/100 g), and the high end (24.15) belonged to Treatment 15 (BT = 132.5^°^C, MC = 22.75/100 g, SPC/MS = 36.25/100 g and MP = 21.25/100 g). Milder extrusion temperatures and a smaller share of nonstarchy ingredients seem to preserve more pronounced yellow tones.

The *b* ^∗^ quadratic model was likewise significant (*p* < 0.05), with *R* ^2^ near 0.74 (Table 2) and a nonsignificant lack of fit confirming its adequacy over the tested domain. As with *L* ^∗^ and *a* ^∗^, BT, the SPC/MS ratio and MP content stood out as the most influential terms in Table 3, along with a few quadratic formulation effects. Figure 2e (BT–MC) shows *b* ^∗^ declining gradually as temperature rises, particularly at lower moisture, plausibly reflecting the breakdown of natural pigments and intensifying browning. Figure 2f shows that raising SPC/MS and MP together also pulls *b* ^∗^ down, again consistent with protein and phenolic compounds contributing to darker pigments that mute the yellow hue. This kind of CO shift driven by thermal browning and pigment breakdown is a familiar feature of extruding fibre‐rich formulations [1, 2, 5].

### 3.3. Effect of BT, MC, SPC/MS Ratio and MP Content on Antioxidant Capacity (DPPH and ABTS)

DPPH inhibition (DPPH%) proved sensitive to the combined severity of formulation and thermomechanical conditions, with values spanning 12.34%–26.28% (Table 3). The peak (26.28%) turned up under high MP (21.25/100 g) and low MC (18.25/100 g) at BT = 132.5^°^C and SPC/MS = 28.75/100 g (Tr9), whereas the trough (12.34%) occurred with low MP (13.75/100 g) and high MC (22.75/100 g) (Tr3). Together, these results confirm that radical‐scavenging capacity hinges both on how much antioxidant material MP itself supplies and on whether processing conditions help release or transform it (Table 1).

DPPH% fit a significant quadratic model, with *R* ^2^ = 0.82 and a nonsignificant lack of fit backing its predictive value (Table 2). ANOVA (Tables 2 and 3) flagged three dominant terms (*p* < 0.05): the linear MC effect (*B*, negative), MP (*D*, positive) and the C × D interaction (SPC/MS × MP, negative); BT and SPC/MS alone were not significant. The coefficients in Table 3 tell the same story: MC pulled DPPH% down (*b*_2_ = −0.233; *p* < 0.05), MP pushed it up (*b*_4_ = +0.291; *p* < 0.05) and the SPC/MS × MP interaction chipped away at MP′s benefit as protein content rose (*b*_34_ = −0.162; *p* < 0.05).

MP′s positive contribution fits what is already known about valorising plant by‐products: *Cucumis melo* peels are a recognised source of dietary fibre and phenolic compounds that feed antioxidant activity, and extrusion itself may help liberate bound phenolics by breaking down the cellular matrix and disrupting noncovalent interactions [23]. The negative MC effect, on the other hand, points to higher moisture curbing how much antioxidant material is generated or released and picked up by DPPH, consistent with high MC lowering melt viscosity and effective shear, which in turn limits structural disruption and, with it, the release of phenolics or browning‐derived reducing compounds [1].

**Figure 3:**
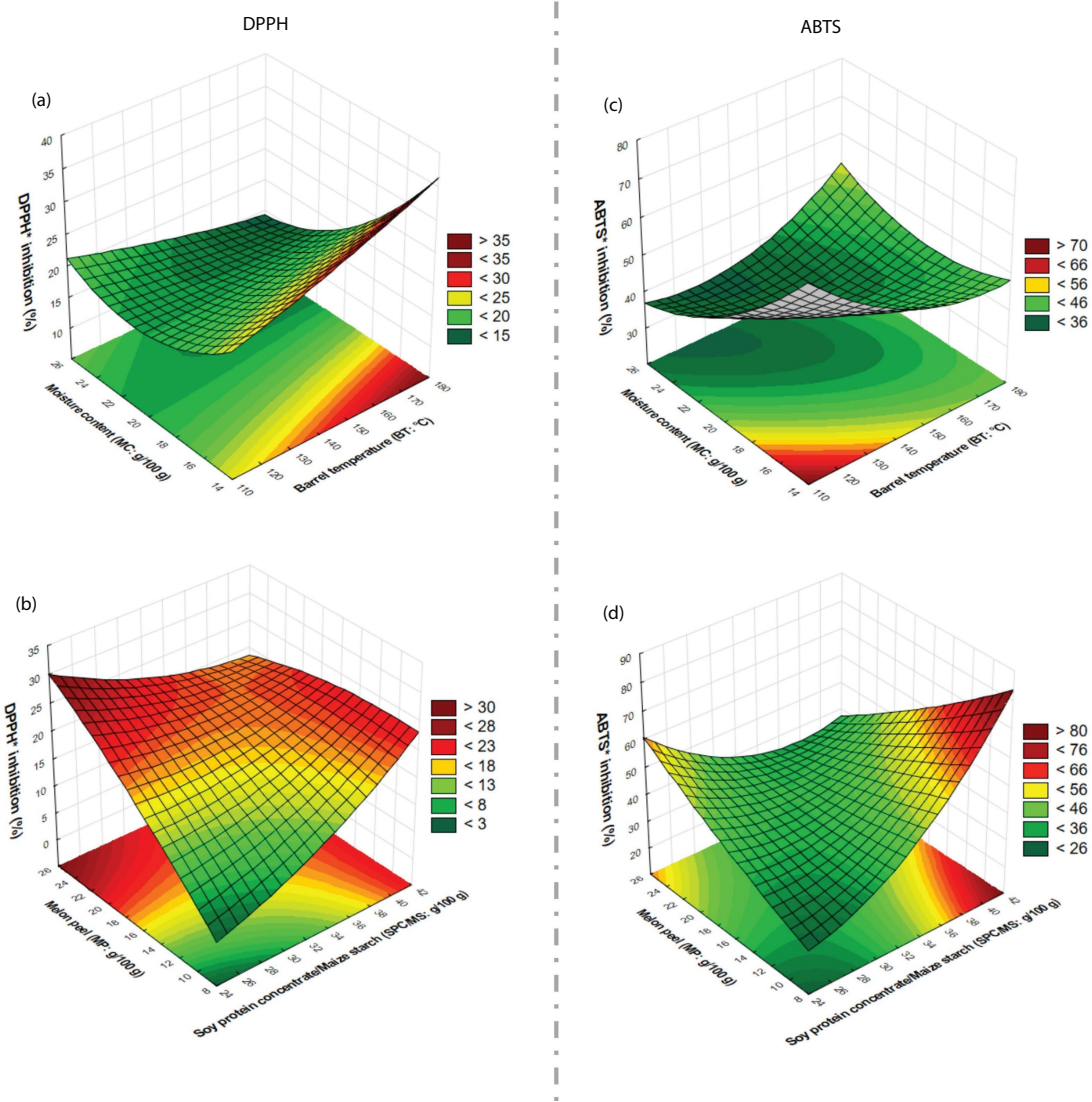
Response surface plots showing the effects of barrel temperature (BT), moisture content (MC), melon peel (MP) and soy protein concentrate/maize starch (SPC/MS) ratio on (a,b) DPPH• inhibition and (c,d) ABTS**•** ^+^ inhibition.

Figure 3a (DPPH vs. BT–MC) shows a gradient dominated by MC, with inhibition climbing as MC drops and getting a further boost in the high BT region consistent with greater thermomechanical severity releasing more antioxidants and/or generating Maillard reaction products with their own radical‐scavenging ability. This echoes findings for cereal–by‐product extrudates elsewhere, where process severity and substrate availability meaningfully shape antioxidant responses [25, 26].

Figure 3b (DPPH vs. SPC/MS–MP) shows DPPH% climbing sharply with MP, though that benefit tapers somewhat as SPC/MS rises, matching the negative C × D interaction. A larger protein share could be doing one of several things: diluting the by‐product′s relative phenolic contribution, driving protein–polyphenol interactions (hydrophobic bonding and hydrogen bonding) that shrink the extractable fraction and thus the measured activity or reshaping the molten matrix′s microstructure in ways that limit how much antioxidant material actually gets released during processing. This kind of trade‐off between by‐product enrichment and protein content has been noted before in functional extrudates, where the biopolymer matrix has a strong say in antioxidant accessibility and how radical‐based assays like DPPH read out [19].

ABTS inhibition (ABTS%) was harder to pin down (31.91%–60.54%, Table 1); the ABTS•^+^ radical clearly responded to the interplay of thermomechanical severity and composition, but far less predictably than DPPH did. Treatment 5 (BT = 132.5^°^C, MC = 18.25/100 g, SPC/MS = 36.25/100 g and MP = 13.75/100 g) posted the top value (60.54%), whereas Treatment 23 (BT = 145^°^C, MC = 20.5/100 g, SPC/MS = 32.5/100 g and MP = 10/100 g) gave the lowest (31.91%) (Table 1). The RSM fit for ABTS% was only modest (*R* ^2^ = 0.53; Table 2), and unlike AD, EI or DPPH, Table 3 turned up no statistically significant coefficients (*p* < 0.05) for this response, meaning the variation seen likely reflects factor combinations or sources of variability the model simply does not capture.

Even without significant coefficients, the response surfaces hint at some underlying tendencies. ABTS values ran a touch higher at lower moisture and intermediate‐to‐high SPC/MS, a pattern plausibly linked to shifts in thermomechanical energy input and to changes within the starch–protein matrix during processing. Given the model′s modest *R* ^2^ (0.53) and the lack of significant regression terms, these observations are best treated as tentative.

Figure 3c,d points in the same direction, suggesting somewhat higher ABTS at lower moisture and intermediate‐to‐high SPC/MS, though again these trends warrant caution given the model′s modest predictive power and the fairly high variability in this particular response. The divergence between DPPH and ABTS outcomes may also simply reflect that the two assays have different selectivity and reaction mechanisms, responding differently depending on the chemical nature of whatever antioxidant compounds are present in the extruded matrix.

This fits with the fact that low MC raises apparent viscosity and effective shear, which pushes up thermomechanical energy and structural disruption conditions that can make antioxidants easier to extract and/or generate more reducing compounds during processing [1, 18]. Meanwhile, the positive SPC/MS effect lines up with proteins acting as substrates for Maillard‐type nonenzymatic browning under thermomechanical stress, a process that can yield melanoidins and other radical‐scavenging compounds, with ABTS•^+^ often picking up on this more readily than other antioxidant assays, depending on the matrix and the exact compounds formed [27].

MP is a plausible source of phenolics and fibre, yet it showed no statistical significance for ABTS% (Tables 2 and 3), likely because, in these formulations, its contribution got overshadowed by the dominant rheological effect of MC and by protein‐ and energy‐driven chemistry. It is also plausible that protein–polyphenol interactions cut into the extractable antioxidant fraction or altered the analytical readout itself, something documented before in higher protein extruded matrices [19].

Set against studies where adding polyphenol‐rich by‐products boosts postextrusion antioxidant activity, the pattern seen here suggests the starch–protein–fibre system may be running two pathways simultaneously: releasing bound phenolics as the plant matrix breaks down and generating browning‐derived antioxidant compounds whose extent depends on moisture and protein content. Peel‐type by‐products in extruded matrices, more generally, have been tied to antioxidant gains that hinge on process severity and formulation, with DPPH and ABTS not always agreeing given their different selectivity [23, 25].

### 3.4. Effect of BT, MC, SPC/MS Ratio and MP Content on the Sensory Attributes of the Extruded Snack

**Figure 4:**
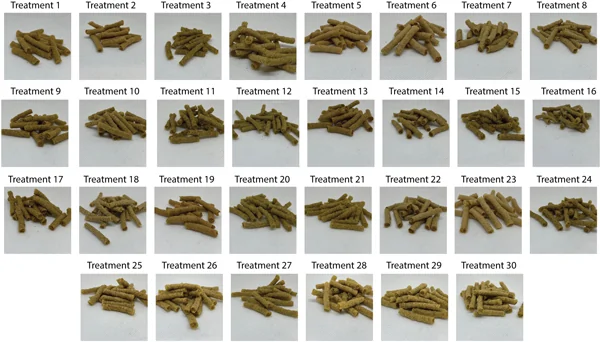
Photographs of the extruded snacks obtained under the different extrusion conditions.

Figure 4 shows photographs of all 30 treatments spanning the tested combinations of BT, MC, SPC/MS ratio and MP content, and the visual differences in expansion, surface TEX, uniformity and CO make clear how strongly formulation and thermomechanical conditions act together. Treatments run at higher temperatures (≈160°C–170°C) with lower moisture generally came out more expanded and porous, with slightly rough surfaces and darker tones most obvious in T28–T30, where the added heat drives more complete starch gelatinisation and builds up specific mechanical energy, raising vapour pressure at the die exit and pushing expansion further.

At the other end, treatments with higher feed moisture and cooler temperatures (≈120°C–130°C) produced more compact, less‐expanded extrudates with smoother, lighter surfaces visible in the earliest treatments (T1–T4) and tied to the melt losing viscosity as water content climbs, which cuts specific mechanical energy and caps expansion. Raising MP content, separately, gave extrudates that were slightly denser and darker, likely a combination of fibre′s structural effect and phenolic compounds accelerating browning under heat. These observations track with earlier work on extruding fibre‐rich agroindustrial by‐products: Mazlan et al. [5] found that plant residues reduce expansion and yield denser structures because fibre interferes with the expandable starch matrix, Ding et al. [1] found that high temperature paired with moderate moisture favours more open, crispy cellular structures and Brennan et al. [2] observed that extrusion intensifies Maillard reactions and caramelisation, darkening the final product.

Across AR, CO, FL, TEX, AP and OA, clear differences emerged from the formulation–process interaction, with overall acceptance settling in a moderate‐to‐high band (OA = 5.20–7.40). The best‐liked formulation (Tr5: BT = 132.5^°^C, MC = 18.25/100 g, SPC/MS = 36.25/100 g and MP = 13.75/100 g) topped every attribute AR (6.60), CO (6.63), FL (7.07), TEX (7.10), AP (7.03) and OA (7.40). The weakest performer, Tr11 (high MC at 22.75/100 g and high MP at 21.25/100 g), dropped sharply in FL (4.77), TEX (4.47) and OA (5.20) (Table 1). Taken together, this suggests that in this MP‐enriched starch–protein system, stacking high moisture on top of high MP hurts sensory perception, TEX and FL especially. That penalty likely traces back to denser, less‐expanded matrices and to fibre‐associated sensory notes becoming more pronounced: a sandy mouthfeel, added hardness or a vegetal aftertaste effect that show up widely as fibrous by‐product levels rise in extruded products [21, 28].

**Figure 5:**
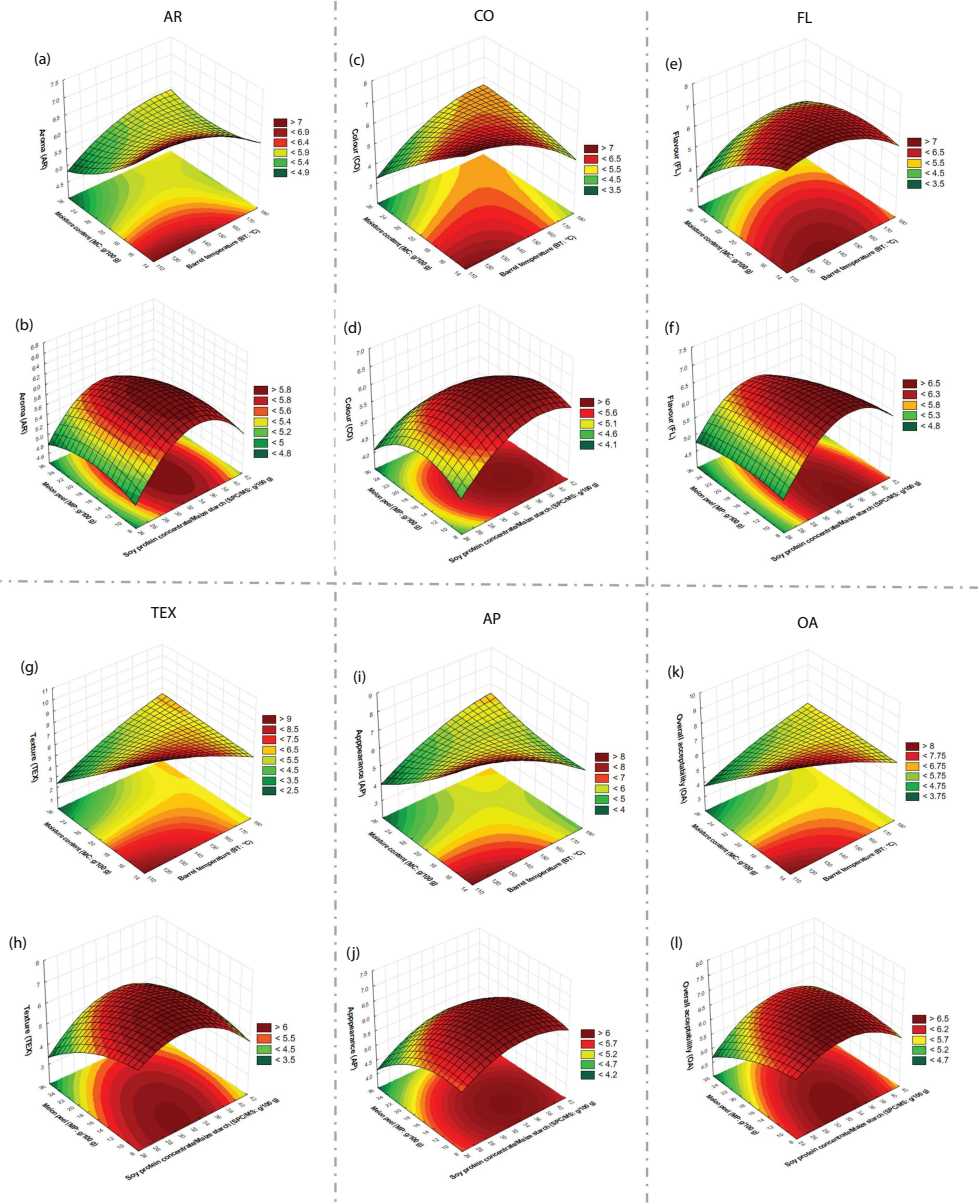
Response surface plots showing the effects of barrel temperature (BT), moisture content (MC), melon peel (MP) and soy protein concentrate/maize starch (SPC/MS) ratio on (a,b) aroma (AR), (c,d) colour (CO), (e,f) flavour (FL), (g,h) texture (TEX), (i,j) appearance (AP) and (k,l) overall acceptability (OA).

All six sensory attributes fit significant quadratic models (Tables 2 and 3), and the pattern across them was remarkably consistent. MC (*b*_2_) carried negative, significant coefficients everywhere (AR −0.041; CO −0.065; FL −0.085; TEX −0.125; AP −0.061; OA −0.091) more moisture, lower scores across the board. This tracks with the BT × MC surfaces in Figure 5a,c,e,g,i,k, where the sweet spots for preference cluster at lower MC and intermediate‐to‐high BT.

SPC/MS (*b*_3_), meanwhile, gave significant positive effects for AR (0.017), CO (0.035) and AP (0.031), but paired with significant negative curvature (*b*_33_) for AR, FL, TEX and OA (OA′s *b*_33_, for instance, sits at −0.025). In other words, sensory quality peaks somewhere in the intermediate‐to‐high SPC/MS range rather than climbing indefinitely with more protein. This tracks with what the literature says more broadly; protein can strengthen body, melt strength and sensory stability up to a point, but push it too far, and hardness rises, or FL shifts due to microstructural and thermal changes [18, 19].

MP (*b*_4_), for its part, carried negative, significant effects on CO, TEX, AP and OA (OA sits at −0.034), matching the SPC/MS × MP surfaces in Figure 5b,d,f,h,j,l, where more MP pulls scores down, TEX and overall acceptance especially. That squares with the structural fibre effects discussed earlier: More fibre means less matrix expansion and denser cells. Higher MP may also darken CO and roughen the surface [1, 21].

The BT × MC interaction (*b*_12_) was positive and significant for CO, TEX, AP and OA (OA at 0.037). In Figure 5, this shows up as a sensory ‘sweet spot’ when BT rises alongside relatively low‐to‐moderate MC; enough heat, in other words, can deliver good CO, AP and TEX without needing to load up on moisture. This tracks with the general understanding of structural development in extrusion, where acceptability comes down to the balance between starch gelatinisation, expansion and cell wall setting during cooling [29]. Overall, acceptability peaked under low MC, low‐to‐moderate MP and intermediate‐to‐high SPC/MS (reflecting *C* ^2^′s curvature), a combination that keeps TEX and FL strong whilst still delivering the nutritional boost the extrudate is meant to provide.

## 4. Conclusion

Taken as a whole, this work shows that formulation was the decisive factor for the product′s nutritional and functional quality, whereas thermomechanical processing conditions chiefly governed how the expanded structure and CO developed. Adding MP raised dietary fibre and antioxidant‐active compounds, lending the snack extra functional value, though pushing this by‐product to high levels also tended to compact the matrix and dent certain sensory scores, pointing to the need for a careful balance between nutritional gain and technological performance. The SPC/MS ratio, meanwhile, left its mark on both chemical composition and sensory perception, and feed MC stood out as the single most influential processing variable for TEX and acceptability, working through its control over melt viscosity and expansion. In short, formulation and extrusion conditions act together to shape microstructure, functional properties and acceptability. The conditions tested here yielded extrudates with good expansion, solid technological stability, an improved nutritional profile and acceptable sensory scores, evidence that MP holds real promise as a functional ingredient for valorising agroindustrial by‐products and that extrusion remains a sound technological route towards healthier, more sustainable snacks in step with current thinking on functional food innovation and the circular economy.

## Author Contributions

Conceptualisation: B.H‐S. and J.R‐M.; methodology: C.E.M‐S. and A.Á‐H.; software: J.M.J‐B.; validation: J.G.T‐U., E.R‐F. and A.Á‐H.; formal analysis: C.E.M‐S. and B.H‐S.; investigation: J.M.R‐R. and A.Á‐H.; data curation: J.M.R‐R., J.M.J‐B. and J.G.T‐U.; writing—original draft preparation: B.H‐S., J.M.J‐B., J.G.T‐U. and J.R‐M.; writing—review and editing: B.H‐S. and J.R‐M.; visualisation: J.R‐M. and B.H‐S.; supervision: J.R‐M.

## Funding

This work was supported by Tecnológico Nacional de México (10.13039/100012725, 13779.22‐P) and SECIHTI (1184001).

## Disclosure

All authors have read and agreed to the published version of the manuscript.

## Ethics Statement

Ethical review and approval were waived for this study, as it involved a noninvasive sensory evaluation of food products with adult volunteer panellists, which does not require approval from an institutional ethics committee under the internal regulations of the Instituto Tecnológico de Tuxtepec.

## Consent

Informed consent was obtained from all subjects involved in the study.

## Conflicts of Interest

The authors declare no conflicts of interest.

## Data Availability

The data that support the findings of this study are available from the corresponding author upon reasonable request.
